# Brace related stress in scoliosis patients – Comparison of different concepts of bracing

**DOI:** 10.1186/1748-7161-2-10

**Published:** 2007-08-20

**Authors:** Hans-Rudolf Weiss, Mario Werkmann, Carola Stephan

**Affiliations:** 1 Asklepios Katharina Schroth Spinal Deformities Rehabilitation Centre, Korczakstr. 2, D-55566 Bad Sobernheim, Germany; 2 Orthomed Scolicare, Orthopedic Technical Services, D-55566 Bad Sobernheim, Germany

## Abstract

**Background:**

The BSSQbrace questionnaire has been shown to be reliable with good internal consistency and reproducibility estimating the stress scoliosis patients have whilst wearing their brace. Eight questions are provided focussing on this topic. A max. score of 24 can be achieved (from 0 for most stress to 24 for no stress). The subdivision of the score values is: 0–8 (strong stress), 9–16 (medium stress) and 17–24 (little stress).

**Study design:**

Two BSSQbrace questionnaires have been posted to 65 patients under brace treatment from our Cheneau light data base. All patients had another kind of brace prior to the Cheneau light. The patients have been asked to rate their stress level using one questionnaire for the current brace and the other for the previous one.

**Results:**

63 Patients (59 girls and 4 boys) returned their fully completed  questionnaires (average age 13,6 years, average Cobb angle 43,7 degrees).  Stress level in the previous brace was 11,04 and in the Cheneau light(r)  13,87. The differences were highly significant in the t-test; t = -4,67; p <  0,001.

**Conclusion:**

The use of the Cheneau light^® ^brace leads to reduced stress and/or impairment for the patients under treatment compared to heavier brace models used so far.

## Background

Quality of life seems to be an important issue for patients with scoliosis [1,2]. Therefore a new instrument has been developed by two physicians and two psychologists working at our centre, to assess the psychological stress scoliosis patients have because of their deformity [3]. This instrument is called the Bad Sobernheim Stress Questionnaire (BSSQ). 8 questions are provided focussing on this topic. The response to each question is scored 0 (most stress) to 3 (least stress). A max. score values of 24 can be achieved.

The following items have been provided:

1. I feel conspicuous by the appearance of my back.

2. I find it hard to show my back in public.

3. I feel embarrassed in situations, in which other people can see my naked back.

4. *I don't feel embarrassed showing my back*.

5. I try not to get too close to other people to avoid that they become aware of my scoliosis.

6. When deciding what kind of clothes to wear or how to wear my hair, I take care my back is hidden.

7. *Scoliosis is a part of me, people have to accept me the way I am*.

8. Because of the scoliosis I avoid activities/hobbies, which otherwise I would love to do.

**Figure 1:**
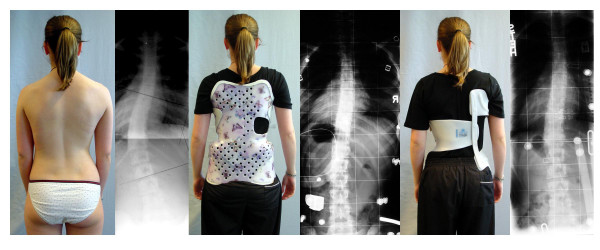
Two braces for one patient. This 14 year old girl with 33° thoracolumbar was not able to wear her initially prescribed Chêneau modification more than 4 hrs./day because of heavy pain for more than 4 months. A new Chêneau light^® ^has been prescribed with similar in-brace correction (of more than 60%) not causing pain anymore. The reduction of material is clearly visible. The BSSQbrace was 5 in her initial brace and 22 in the Chêneau light^® ^brace.

Not only scoliosis itself but also conservative treatment may contribute to a decreased quality of life. The impact of the treatment procedures, especially with respect to brace treatment (Fig. 1.) on quality of life has not been investigated with special questionnaires until 2005, when the first attempts where undertaken to focus on the problems adolescents might have because of brace wearing [4-6].

Derived from the BSSQ, a questionnaire was developed to estimate the psychological stress adolescent scoliosis patients have because of the brace they wear. This BSSQbrace questionnaire has already been tested previously [4,5].

As reported in a previous study, in brace the patients seem to have more stress than just because of their deformity. The in-brace values of the BSSQbrace showed to be lower than the out-brace values related to deformity [5].

In a series of AIS brace patients the BSSQbrace questionnaire has shown to have a good score distribution, and to be internally consistent and reproducible [7]. It can be used to measure the coping strategies a patient uses and the impairment a patient feels to have, whilst wearing a brace. The original German version of this questionnaire as well as the English (still not validated) version are documented as here additional files 1 and 2.

Different bracing concepts are used today for the treatment of scoliosis. The plaster cast method worldwide seems to be the most practiced technique at the moment. CAD (Computer Aided Design) systems are on the market which allow brace adjustments without plaster [8,9]. The latest development however, is the use of the ScoliOlogiC™ off the shelf system enabling the orthopedic technician to construct a light brace (Fig. 1.) for scoliosis correction from a variety of pattern specific shells to be connected to an anterior and a posterior upright [9,10]. This „Chêneau light^®^“ brace, developed according to the Chêneau principles, promises a reduced impediment of quality of life in the brace. To investigate as to whether our hypothesis is right that brace related stress in this brace is less than for other braces, this study has been performed.

## Methods

Two BSSQbrace questionnaires (Additional file 1 and 2) have been posted to 65 patients under brace treatment from our Cheneau light data base. All patients had another kind of brace prior to the Cheneau light and because of that were experienced brace users for at least 6 months. The patients have been asked to rate their stress level using one questionnaire for the current brace and the other for the previous one.

The Student's t-test for dependent samples was performed using Winstat^® ^Software in order to test the hypothesis that the BSSQbrace  values rated by the patients are different for the different braces used.

Of the 63 patients (59 girls and 4 boys) returning the questionnaire 58 had some Chêneau brace or modification before the Chêneau light^® ^has been adjusted (31 from outside and 27 from one of the two bracing companies serving our center), 1 wore a Boston brace, 2 a so called Chêneau-Boston-Wiesbaden brace, 1 a Wilmington brace and 1 a SpineCor.

## Results

63 Patients returned their fully completed questionnaires. Average age  was 13,6 years (Range 10 - 17 years; SD 1,4), average Cobb angle 43,7  degrees (Range 17 - 75°; SD 16,1). Stress level in the previous brace was  11,04 (Range 1 - 23; SD 5,7) and in the Cheneau light 13,87 (Range 4 - 24;  SD 5,3). The differences were highly significant in the t-test; t = -4,67; p  < 0,001.

The BSSQbrace results for the individual questions comparing the previous brace with the Chêneau light^® ^brace are documented in Table 1.

**Table 1:** The BSSQbrace results for the individual questions comparing the previous brace with the Chêneau light^® ^brace

**Question Nr.**	**Previous Brace**	**Chêneau light Brace**
1	0,78 (SD 0,97)	1,34 (SD 1,01)
2	1,21 (SD 1,02)	1,60 (SD 0,92)
3	1,08 (SD 1,02)	1,44 (SD 0,99)
4	1,46 (SD 1,10)	1,71 (SD 1,04)
5	1,46 (SD 1,09)	1,92 (SD 0,99)
6	0,71 (SD 0,96)	1,03 (SD 1,02)
7	2,21 (SD 0,95)	2,37 (SD 0,83)
8	2,33 (SD 1,14)	2,60 (SD 0,68)

Three immature patients had curvatures of less than 20 before being braced repeatedly, but they all had curvatures of more than 20° at the start of their treatment.

## Discussion

Braces in Adolescent Idiopathic Scoliosis (AIS) treatment seem to produce stress [11-14], however there is controversy whether health related quality of life issues of brace treated adolescents are affected negatively [1,13,15,16]. AIS has to be regarded as a chronic condition that affects the body configuration of the adolescent, consequently leading to alterations in lifestyle. The impact of the brace to the self and body image of the adolescent is reported as the main contributory factor for stress production [18-26].

**Figure 2:**
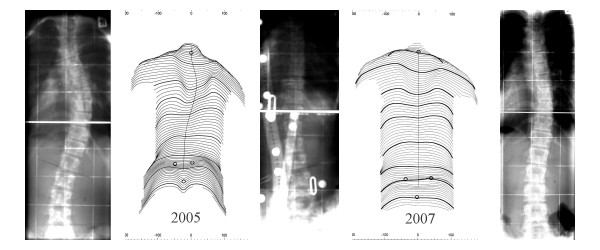
Overcorrection of a thoracic curve from 38° to – 14° in a 11 year old premenarchial girl treated with a T2 „Chêneau light^®^“ model. After 2 years of treatment she has 19° without brace and looks quite compensated in the scans.

Today there is evidence that brace treatment enabled to prevent curve progression [8,17-20] and help to reduce the prevalence of surgery[21,22], but even more than that. Modern bracing concepts clearly do have an impact on the clinical appearance of the patient [23-26]. Even if a curve may progress sometimes under a certain brace treatment clinical appearance may improve drastically [26]. Usually, however, the clinical improvement comes along with an improvement of Cobb angle (Fig. 2.) [23-26]. Clinical improvements have been found achievable in the normal range of bracing indications with curves less than 45° [27] but also in curves bigger than that [28].

We do know that compliance is crucial to the outcome of brace treatment [29] and that there are ways to improve compliance. One way to improve is certainly using the appropriate psychological intervention. If the physician is not convinced that braces do work, so how can he instruct and guide the patient properly? How can the patient believe being braced should be worthwhile? How can even a prospective randomized study on bracing only including so called "non-believers" [29] lead to a conclusion other than: "The brace does not work!" As Landauer [30] recently has pointed out: "Failure has one name: Physician!"

Therefore it is essential to ease the psychological burdon the patient has to bear, by using the appropriate psychological intervention for a patient under brace treatment, a conservative specialist in this field has to have the appropriate skills for.

The other way to improve compliance according to this study may be the reduction of material, in other words: "Make the braces smaller!" When patients experience less stress in the brace we may assume that compliance improves. At this very moment this of course is only an unproven assumption and we are not able to provide enough data on brace wearing times. However in the near future we will look at the relation of reported/prescribed brace wearing times more closely which are introduced into our prospective Chêneau light^® ^database.

**Figure 3:**
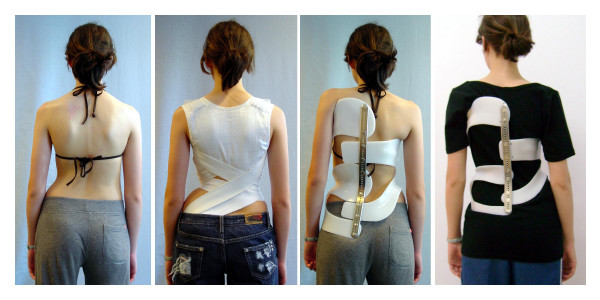
Patient feels very tired in the SpineCor adjusted in Switzerland (Middle left) because she recognized much axial load and has difficulties to breath! No change of decompensation with 41° without and 43° in the SpineCor. Middle right: „Try on“ brace recompensating to the middle and on the right „ready to show“ the final form showing a clear overcompensation to the left. The patient feels less stressed in this brace than in the SpineCor she was treated with before: BSSQbrace SpineCor 7 and BSSQbrace Chêneau light^® ^10.

Interestingly not only the usual hard braces have been found to induce more stress than the Chêneau light^®^, but also one patient under SpineCor treatment reported to have more stress in the SpineCor than while wearing the Chêneau light^® ^(Fig. 3.). In this case, however, it was surely not the material reduction but the compression experienced and the impairment of breathing felt by the patient. However this is not very common in patients under SpineCor treatment [31].

It certainly would have been interesting to compare the different brace types with the help of the BSSQbrace questionnaire, however there were only few braces other than Chêneau types in this study and therefore not enough numbers to make a statistical comparison or to draw conclusions. Furthermore there is a great variety in the quality of the different braces also in the Chêneau group which makes it hard to compare different braces just on the basis of the braces names.

**Figure 4:**
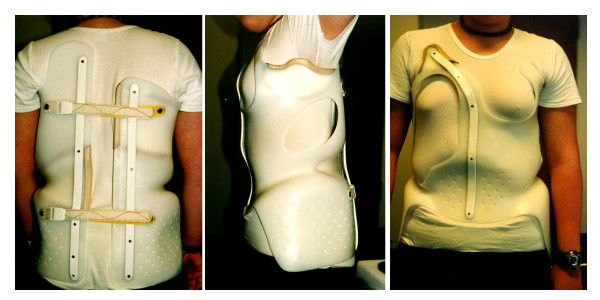
CBW (Chêneau Boston Wiesbaden) brace combining the application of some Chêneau pressure areas with the Boston style brace which is being closed dorsally. The left picture shows the rear view, the middle the side view and the right picture the front view.

Boston or Wilmington braces are well recognized in international literature, however the Chêneau-Boston-Wiesbaden brace (CBW) is not known internationally. Therefore I would like to demonstrate a CBW brace on figure 4 just to give an example. The brace on figure 4 however is not the brace of a patient from this study.

## Conclusion

The use of the Cheneau light brace leads to reduced stress and/or impairment for the patients under treatment compared to heavier brace models used so far.

More studies are needed to enlighten the different facets of compliance in order to improve strategies able to increase brace wearing time.

## Competing interests

The first author is currently applying for a patent relating to the Chêneau light^® ^brace. None of the authors has received any reimbursements, fees, funding, or salary related to the content of this paper.

## Authors' contributions

HRW: Study design, data acquisition, analysis and interpretation of data, preparation of the manuscript, corresponding author.

MW: Data acquisition.

CS: Data acquisition, data base.

## Supplementary Material

Additional file 1 BSSQbrace original German version. Original sheet the patients had to rate their stress level with. Click here for file

Additional file 2 BSSQbrace English version (reliability not tested). Translated version for the documentation within this study. Click here for file
